# Biological Evaluation of a Siliconized Analog of Clofibrate (Silafibrate) in Rodents

**Published:** 2013

**Authors:** Mojtaba Ziaee, Mohammad Ali Eghbal, Jafar Rahmani, Mohammad Ghaffarzadeh, Arash Khorrami, Alireza Garjani

**Affiliations:** a *Young Researchers Club, Tabriz Branch, Islamic Azad University, Tabriz, Iran.*; b *Drug Applied Research Center, Tabriz University of Medical Sciences, Tabriz, Iran.*; c *Department of Pharmacology and Toxicology, Faculty of Pharmacy, Tabriz University of Medical Sciences, Tabriz, Iran.*; d *Department of Clinical Sciences, Tabriz Branch, Islamic Azad University, Tabriz, Iran.*; e *Chemistry and Chemical Engineering Research Center of Iran, Tehran, Iran.*; f *Student Research Committee, Faculty of Pharmacy, Tabriz University of Medical Sciences, Tabriz, Iran. *

**Keywords:** Clofibrate, Siliconizedanalog, Silafibrate, Hypolipidemicdrug, Toxicity

## Abstract

Silicon is the element very similar to carbon, and bioactive siliconized compounds have therefore received much attention. Siliconization of a compound enhances its biological activities. In the present study the hypolipidemic effect and toxicity of clofibrate and its siliconized analog, silafibrate, were compared. The experiments were performed in hypercholesterolemicWistar rats. Animals received high fat diet with 62.75% normal chow, 2% cholesterol, 0.25% cholic acid, 15% lard oil, 10% wheat flour and 10% sucrose.Silafibrate(40 mg/kg/day) produced a predominant reduction in the serum levels of total cholesterol (28.4%, p < 0.001), triglycerides (62%, p < 0.0001) and low-density lipoproteins (27%, p < 0.001) being more effective than the reference drug clofibrate (20%, 40%, 14.5%; p < 0.05). Similarly, it increased the total antioxidant levels in serum by 40% (p < 0.05). Simultaneously, treatment with silafibrate also reduced the malondialdehyde(MDA) concentration by 41% (p < 0.05). LD_50 _of silafibrate, given orally,was greater than 2000 mg/kg body weight inalbino mice while LD_50_ for clofibrate was calculated to be 1220 mg/kg. Thirty-day subacute toxicity was also evaluated with oral daily dose at 25, 50 and 100 mg/kg body weight in Wistarrats. No significant changes in body weight, food intake, behavior, mortality, hematology, blood biochemistry, vital organ weight were detected. The results of this study indicate that the effectiveness and safety of thehypolipidemic drug, clofibrate, were enhanced remarkably by replacing chlorine atom in its phenoxy ring with trimethylsilyl.

## Introduction

Hyperlipidemia is the major underlying cause of atherosclerosis and atherosclerosis-related diseases such as coronary heart disease, peripheral vascular complications, and ischemic cerebrovascular events (1). A large number of studies have demonstrated that the elevated level of triglyceride (TG) and low concentration of high-density lipoprotein (HDL)-cholesterol in serum are among the major risk factors for atherosclerotic vascular disease (2). Fibrates, peroxisome proliferator-activated receptor-α (PPAR-α) agonists, are hypolipidemic drugs that efficiently lower serum triglyceride and increase HDL cholesterol (HDL-C) levels. These drugs also reduce low-density lipoprotein cholesterol (LDL-C), which is correlated with increased risk of atherosclerosis (3). Fibrates are the first-line drugs for the treatment of primary hypertriglyceridemia. Their effect is mediated through their interaction with PPAR-α which results in the stimulation of fatty acid oxidation, increased lipoprotein lipasesynthesis, and reduced expression of apolipoprotein C-III (4). Fibrates differ in their potency, and it seems a structure–activity relationship (SAR) exists (5). Bezafibrate, ciprofibrate, and fenofibrate are halogenated derivatives of clofibrate as the parent analog and contain phenoxy-2-methyl-2-propanoic acid chain in their structures (6). It is obvious that the high lipophilicity is required for the effectiveantidyslipidemic actions of fibrates (7).

Silicon-based drugs are now being developed, and silicon-containing compounds have entered human clinical trials (8). Siliconizedmedicinal compounds have been shown to enhancetheir biological activity and improvepharmacological profiles in comparison to their corresponding isosteres (8, 9). Siliconization of current drugs changes the geometric and electronic aspects and therefore the size, shape, conformational behavior, chemical reactivity and lipophilicity of the molecule (10). This might in turn alter the interaction with their respectivereceptor and thus alter the pharmacodynamics of the drug. The metabolism of the drug may change and therefore also metabolism-related toxicity (10).

**Figure 1:**
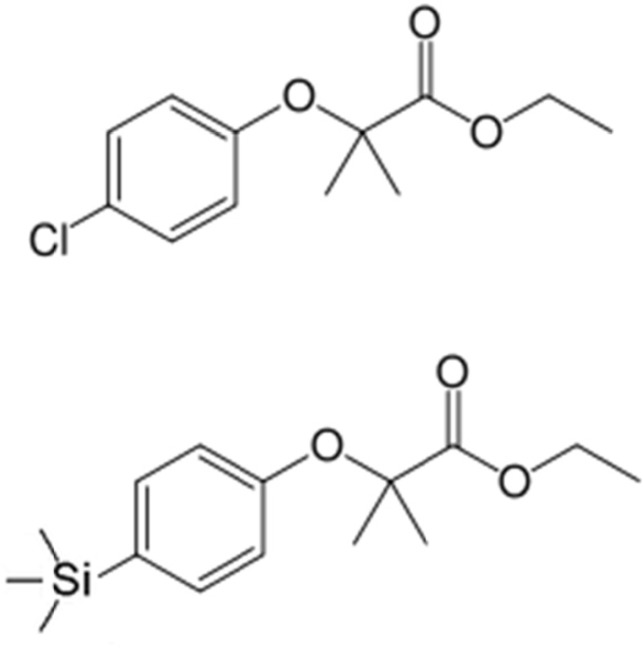
Chemical structure of clofibrate (upper panel) and silafibrate (lower panel)

We have previously synthetized the novel analog by the replacement of chlorine atom in phenoxy ring of clofibrate with trimethylsilyland this new analog was named silafibrate (ethyl-2-methyl-2-(4-(trimethylsilyl)phenoxy)propionate) (Figure 1) (11).

To search foran effective and a safe hypolipidemic drug, in the present study we evaluated the biological activity of silafibrate in hyperlipidemic rats and compared its effects with those of clofibrate. The acute and subacute toxicity of the oral administration of the new analog were also assessed in this study.

## Exprimental

*Chemicals*

Silafibrate used in this study was synthesized and identified in Chemistry and Chemical Engineering Research Center of Iran. Clofibrate was a giftfromZahraviPharmaceutical Company (Tabriz, Iran) with the European Pharmacopoeia specifications. All other chemicals and solvents used in experiments were of highest purity and purchased locally. 

*High-fat diet*

A high-fat diet (HFD) suggested by Roberts and coworkers (12) was used in this study with some modifications. The high-fat pellet diet contained standard SahandNiroo (Tabriz-Iran) rodent chow powder (62.75%), cholic acid (0.25%), cholesterol(2%), and lard oil (15%), wheat flour (10%), and sucrose (10%).

*Experimental protocol*

Male Wistarrats weighting 180200- grams were randomly allocated into six groups.Each group consisted of six animals. The first group received a normal rodent chow (normal control) *ad libitum*, while the other groups (groups 2–6) were fed by high fat diet (HFD) with free access to food and water for 5 weeks to induce hyperlipidemia. After induction of hyperlipidemia, at day 7, silafibrate or clofibrate were given orally at doses of 20 and 40 mg/kg in 0.5% sodium carboxymethylcellulose (CMC) per day. Normal controlrats received vehicle (0.5% CMC) at the same volume throughout the experimental period.At the end of the experiments, the rats were fasted for 12 h and then anaesthetized by pentobarbital (50 mg/kg, IP). Blood samples were collected from abdominal aorta and transferred into two centrifuge tubes. One tube containing EDTA (3 mmol/L or 0.1% final conc.) and the other one without any additive were centrifuged to obtain plasma and serum, respectively. Serum was separated by centrifugation at 3000 rpm for 10 min for the measurement of biochemical parameters.

*Serum biochemical analysis*

Serum concentrations of total cholesterol (TC), triglycerides (TGs) and HDL cholesterol (HDL-C) were measured by enzymatic colorimetric methods using commercially available kits (Pars Azmoon Laboratories, Iran). The assays were performed according to the manufacturer’s instructions. All samples were analyzed in duplicate. The concentration of serum LDL–cholesterol was determined according to Friedewald’s formula by the following equation: LDL = TC − (HDL + 0.2TG) (15). 

*Determination of lipid peroxidation and total antioxidant status*

Serum malondialdehyde (MDA) level, the end-product of lipid peroxidation, as a marker of lipid peroxidation and oxidative stress was measured through reaction with thiobarbituric acid (TBA) as a TBA reactive substance (TBARS) to produce a pink colored complex. Then, its fluorescence intensity was measured at 586 nm with excitation at 560 nm by a spectrofluorometer (Kontron, model SFM 25A, Italy) (13).Measurement of total antioxidant status (TAS) in serum was performed by colorimetric method with commercial kit (RANDOX kits, RANDOX Laboratory, UK), on an automatic analyzer (Abbott model Alcyon 300, USA).

*Acute toxicity*

Eighty mice of either sex were divided according to the gender and housed in standard clear plastic cages (10 micein each). They were maintained in standard environmental conditions (22–24 °C; 12:12 h dark/light cycle). Mice had free access to water and normal lab food, except for a short fasting period before the treatment with the single dose ofsilafibrate. Silafibrate was suspended in 0.5% CMC in distilled water. A control group received the vehicle (0.5%; CMC) orally. Treatment groups were orally gavagedwithsilafibrate at doses of 500, 1000, 1500 and 2000 mg/kg according to Organization of Economic Cooperation and Development (OECD) methods (14). Animals were closely observed for 14 days for toxic manifestations such asbody weight changes, abnormal motor activity, and alteration in water or food intake, gross morphological changes, behavioural changes and mortality.

*Subacute toxicity*

Healthy male and female Wistarrats weighting between 185–200 g (n = 80, 10 per sex) were used in this study. The animals were obtained from the animal house of Tabriz University of Medical Sciences and maintained under a controlled ambient temperature of 22- 24 °C, with 40 ± 10% relative humidity and a 12:12 light/dark cycle. They were fed with standard laboratory chow and tap water *ad libitum*. The care and handling of all animals were in accordance with the internationally accepted guidelines for the use of animals and accordance with the guidelines of the Tabriz University of Medical Sciences, Tabriz, IR Iran, regarding the care and use of laboratory animals (National Institutes of Health Publication No 85-23, revised 1985).

*Observation and examination methods*

The experiments were performed according to Organization for Economic Co-Operation and Development (OECD) Test Guidelines with few modifications (14). Silafibrate was suspended in 0.5% CMC and administered daily by gavage for 30 days at doses of 25, 50 and 100 mg/kg of body weight while the control rats received the vehicle (0.5% CMC). On the day 30 the pathologic evaluations were conducted.Body weight was recorded weekly and food consumption and water intake were monitored daily. Animals were observed for the signs of abnormalities during the treatment period. At the end of the treatment, animals were fasted overnight, but allowed access to water *ad libitum*. They were then anesthetized with pentobarbital (80 mg/kg, IP), blood samples were collected from the abdominal aorta and transferred into two tubes for hematological and biochemical studies, with and without anticoagulant ethylenediaminetetraacetic acid (EDTA) (15), respectively.

*Hematological and biochemical analysis*

The hematological analysis including white blood cell (WBC) and red blood cells (RBC) counts, mean corpuscular volume (MCV), mean corpuscular hemoglobin (MCH) and mean corpuscular hemoglobin concentration (MCHC) were performed using an automatic hematological analyzer (Coulter, Beckman Coulter Company, United States). The differential leukocyte count was performed with optical microscopy after staining and, in each case, 100 cells were counted. For the biochemical analysis, blood was centrifuged at 3000 rpm for 10 min to obtain serum, which was stored at −20 °C until the following parameters were determined: glucose; blood urea nitrogen (BUN); creatinine; aspartate aminotransferase (AST); alanine aminotransferase (ALT); alkalinephosphatase (ALP); total cholesterol (TC); triglycerides(TG); high-density lipoproteins (HDL). The concentrations of TC, HDL-C and TG were measured by enzymatic colorimetric methods with commercial kits (Cholesterol CHOD-PAP and Triglycerides GPO-PAP; Pars Azmoon, Iran) on an automatic analyzer (Abbott, model Alcyon 300,USA) (16). Serum LDL-C was calculated according to the Friedewald equation (17). The assays were performed according to the manufacturer’s instruction. All samples were measured in duplicate.

*Organs weight and morphology*

The animals were euthanized with an excess of pentobarbital (80 mg/kg, IP), and then necropsy was performed (n = 10/group/sex) in order to analyze the macroscopic external features of the heart, liver, spleen, kidney, brain and reproductive organs (uterus and ovary or testicle, prostate, epididymis, seminal vesicle and vas deferens). These organs were carefully removed and weighed individually. Organ weights were expressed as wet weight. 

*Statistical analysis*

Data are expressed as Means±SEM. Differences between groups were tested using one-way ANOVA followed by Tukey›s post hoc comparison test. A probability value less than 0.05 was considered as statistically significant. 

## Results

*Hypolipidemic effect*

**Figure 2:**
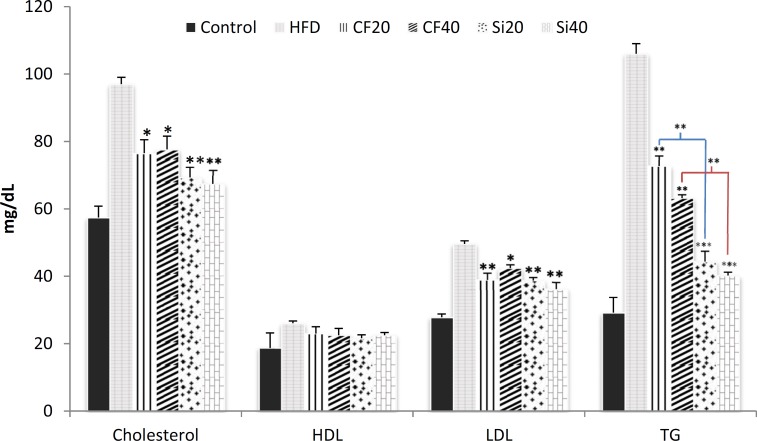
Effects of oral administration of clofibrate and its novel analog,silafibrate,for 7 successive days (20, 40 mg/kg/day) on serum lipid profiles as triglycerides (TG), total cholesterol, HDL, and LDL in hypercholesterolemic rats. Data presented as mg/dl. Data are expressed as mean±SEM. n=7. *p<0.05, **p<0.001, ***p<0.0001 compared with hypercholestrolemic rats using ordinary ANOVA test. HFD= hypercholesterolemic rats; Cf20= clofibrate 20 mg/kg, Cf40= clofibrate 40 mg/kg Si20 = silafibrate20 mg/kg, Si40= silafibrate 40 mg/kg

High fat diet significantly increased the level of serum lipids compared to control (p < 0.001). The results showed that feeding rats with the high-fat diet for 5 weeks significantly elevated the serum level of total cholesterol, triglycerides, and LDL by 68%, 263% and 78%, respectively (Figure 2).Moreover, the induction of hyperlipidemia significantly increased serum level of HDL by 40%. The oral administration of silafibrate (20 mg/kg/day) for 7 days to the hyperlipidemic rats significantly reduced the serum level of total cholesterol from 972± mg/dl in hyperlipidemic rats to 69.33±, triglycerides from 106 ±3 mg/dL to 44.43± and LDL from 49.60.9± mg/dL to 38.71± (p < Meanwhile, clofibrate at the same dose (20 mg/kg/day) also decreased the total cholesterol to 76.54± mg/dL, triglycerides to 72.73± mg/dL and LDL to 23.02± mg/dL (Figure 2). Oral treatment of hyperlipidemic rats with silafibrate at dose of 40 mg/kg for 7 days lowered the serum level of total cholesterol to 67.44± mg/dL, LDL level to 36.22± mg/dL and triglycerides to 40.21± mg/dL while clofibrate at the same dose reduced the serum level of total cholesterol to 77.64± mg/dL, LDL level to 42.41± mg/dL and triglycerides to 63.21± mg/dL respectively (p < 0.001) (Figure 2). Significant reduction of serum cholesterol and triglyceride levels were observed in all silafibrate and clofibrate treated groups (p < 0.001). However, silafibrate was more effective (p < 0.05) than clofibrate.There were no significant changes in the serum level of HDL in the treated groups.

*Serum MDA and total antioxidant status*

**Figure 3:**
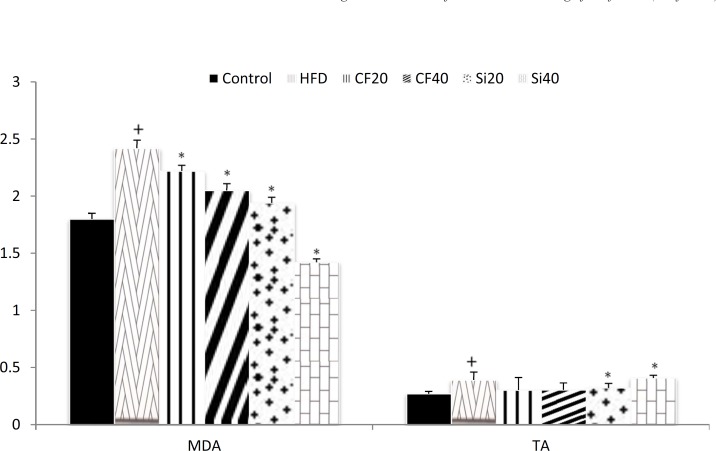
Effects of oral administration of clofibrate and its novel analog,silafibrate,(20, 40 mg/kg/day) on serum MDA (nmol/ml) and total antioxidant concentrations (U/L)in hypercholesterolemicrats. Data are expressed as mean±SEM. n=7. +p<0.05 compared with normocholesterolemic rats. *p<0.05 compared with hypercholestrolemic rats using ordinary ANOVA test and LSD post hoc test. HFD= hypercholesterolemic rats; Cf20= clofibrate 20 mg/kg, Cf40= clofibrate 40 mg/kg Si20 = silafibrate20 mg/kg, Si40= silafibrate40mg/kg

The changes in the serum levels of MDA are shown in Figure 3. High fat diet significantly increased the level of serum MDA (p < 0.05) and both clofibrate and silafibrate at 20 and 40 mg/kg produced considerable reduction in the level of MDA compared with hyperlipidemicrats (p < 0.05). Serum total antioxidant status (TAS) level was significantly elevated after the induction of hypercholesterolemia (p < 0.05). Oral treatment with silafibrate20 and 40 mg/kg/day for 7 days significantly increased TAS level (p<0.05)however,clofibrate did not exert a significant effect (Figure 3). 

*icity of silafibrate in mice*

No deaths were observed during 14 days of observation after the male and female mice were orally given silafibrate at doses of 50, 100, 250, 500, 1000, 1500 and 2000 mg/kg. Acute oral treatment with silafibrate at doses up to 2000 mg/kg did not produce any sign of toxicity or death in mice during period of observation. Some adverse effects such as hypoactivity, trembling and asthenia were observed in the 1500 and 2000 mg/kg treated groups. These symptoms gradually alleviated and disappeared within 48h, and there was also no treatment-related mortality in the animalsgiventhese doses. Therefore, the LD50 could not be calculated byoral route, and it is possibly higher than 2000 mg/kg. LD50 for clofibrate has been calculated to be 1220 mg/kg in mice previously (18).

*Subacute toxicity of silafibrate in rats*

*General signs*

No deaths or significant changes in general behavior or other physiological activities were observed during theoral administration of silafibrate at doses of 25, 50 and 100 mg/kg/day for 30 days. All the control and treated rats appeared consistently healthy throughout the study period. No deaths were recorded either in the controls or the treated rats suggesting that the LD50 for chronic oral dosing with silafibrate was much higher than 100 mg/kg per day.

*Body weight changes*

Average pre- and post-treatment bodyweights were measured and the changes in weight were calculated at the end of 30 days study for silafibrate treated and control groups. As summarized in Table 1, no significant changes were recorded in body weight and daily food intake in the treated rats as compared to the control (0 mg/kg).Both the control and silafibrate treated rats appeared consistently healthy throughout the 30-day period of the study. 

**Table 1:** Effects of silafibrate on body weight and food intake of rats

Items	Silafibrate (mg/kg BW)							
	0		25		50		100	
	M	F	M	F	M	F	M	F
Initial weight (g)	190±8	188±6	189±4	186±4	192±5	196±4	193±5	191±5
Weight at week1(g)	215±11	210±9	212±14	208±8	216±14	206±11	211±10	201±14
Weight at week2(g)	235±14	229±12	239±15	227±13	233±19	229±14	237±15	226±11
Weight at week3(g)	259±16	246±14	256±14	257±12	264±16	245±13	256±17	251±16
Weight at week4(g)	282±16	263±17	279±18	261±14	277±18	260±15	274±13	263±13
Food intake (g)	622±49	583±37	618±32	579±44	631±39	589±36	623±45	587±31

Values are means±SEM (n = 80, 20 per group, in each group 10 per sex).M, male; F, female

*Morphological parameters*

The wet tissue weight of rats’ organs obtained from treated and control groups in male and female rats are presented in Tables 2 and 3. The chronic oral administration of silafibrate over 30 days caused no significant changes in the weights of the organs.The liver-body weight ratios in the control and 25, 50 and 100 mg/kg/day silafibrate-treated rats indicated no induction of hepatomegaly (Tables 2, 3). The sex organs weights in treated rats had no significant difference with control groups (Tables 2, 3). The macroscopic analysis of the target organs of the treated animals did not show significant changes in color and texture when compared with the control group (data not presented).

**Table 2:** Effect of the silafibrate (0, 25, 50 and 100 mg/kg) on absolute organ weight in female Wistar rats treated via oral route for 30 consecutive days

Organ	Control	Silafibrate 25mg/kg	Silafibrate 50mg/kg	Silafibrate 100mg/kg
Brain	1.13 ± 0.04	1.11 ± 0.03	1.12 ± 0.03	1.11 ± 0.04
Liver	8.21 ± 0.53	8.26 ± 0.45	8.35 ± 0.28	8.77 ± 0.26
Kidney	1.79 ± 0.04	1.82 ± 0.02	1.8 ± 0.03	1.78 ± 0.04
Spleen	0.88 ± 0.07	0.90 ± 0.06	0.85 ± 0.06	0.89 ± 0.07
Heart	0.93 ± 0.05	0.91 ± 0.07	0.88 ± 0.06	0.92 ± 0.05
Lung	1.20 ± 0.04	1.14 ± 0.06	1.09 ± 0.03	1.19 ± 0.05
Ovary	0.049 ± 0.003	0.047 ± 0.002	0.051 ± 0.002	0.049 ± 0.003
Uterus	0.584 ± 0.066	0.573 ± 0.082	0.506 ± 0.061	0.536 ± 0.054

Values represent the mean±S.E.M. (n = 10/group)

**Table 3:** Effect of the silafibrate (0, 25, 50 and 100 mg/kg) on absolute organ weight in male Wistar rats treated via oral route for 30 consecutive days

Organ	Control	Silafibrate 25mg/kg	Silafibrate 50mg/kg	Silafibrate 100mg/kg
Brain	0.03 ± 1.24	0.04 ± 1.25	0.02 ± 1.26	0.04 ± 1.24
Liver	0.37 ± 10.3	0.50 ± 10.5	0.49 ± 10.8	0.64 ± 10.8
Kidney	0.04 ± 1.93	0.05 ± 1.97	0.04 ± 1.96	0.04 ± 1.95
Spleen	0.07 ± 0.88	0.06 ± 0.90	0.06 ± 0.85	0.07 ± 0.89
Heart	0.05 ± 1.13	0.08 ± 1.11	0.07 ± 1.18	0.05 ± 1.2
Lung	0.09 ± 1.62	0.09 ± 1.64	0.08 ± 1.69	0.09 ± 1.69
Testis	0.08 ± 2.1	0.1 ± 1.97	0.05 ± 1.95	0.06 ± 2.04
Prostate	0.1 ± 0.67	0.09 ± 0.69	0.1 ± 0.68	0.08 ± 0.69
Vas deferens	0.06 ± 0.134	0.07 ± 0.137	0.04 ± 0.133	0.05 ± 0.136

Values represent the mean±S.E.M. (n = 10/group).

*Effect of silafibrate on the hematological and biochemical blood parameters of the rats*

The haematological parameters, including RBC, haemoglobin, MCV, MCH, MCHC and WBC and the blood biochemical parameters, includingcreatinine and glucosedid not vary between control and treated rats (Tables 4, 5).The results indicated that all hematological parameters measured (white blood cells, lymphocytes, monocytes, granulocytes, red blood cells, red blood cell distribution width, hemoglobin, mean corpuscular hemoglobin, mean corpuscular volume) remained within the physiological range throughout the treatment period in 30 days (Table 4). Subacute oral administration of silafibrate (daily for 30 days) did not cause any significant changes in biochemical parameters including total protein, albumin, creatinine, blood urea nitrogen (BUN) and uricacid (Table 5). However, activity markers of liver such as ALP, AST and ALT were increased in dose related manner but the changes were not statistically significant (Table 5). The levels of triglycerides and cholesterol were significantly decreased in the silafibrate-treated rats after 30 days of treatment (p < 0.05) compared to the control rats treated with the vehicle.

**Table 4:** Effect of the silafibrate (0, 25, 50 and 100 mg/kg) on the hematological blood parameters of rats after 30 consecutive days of oral administration

Parameters (units)	Silafibrate (mg/kg BW)							
	0		25		50		100	
	M	F	M	F	M	F	M	F
Erythrocytes (x106/μL3)	7.7±0.3	7.4±0.2	7.6±0.4	7.4±0.4	7.5±0.5	7.3±0.4	7.4±0.5	7.2±0.5
Leucocytes (×109/l)	8.0 ± 1.3	11.2±2.1	7.8 ± 1.1	10.7±0.8	8.1 ± 0.9	11.4±1	7.9 ± 1.2	11.3±1.4
Hemoglobin (mg/dL)	13.8±0.6	14.2±0.8	13.7±0.5	14.0±0.8	13.6±0.9	13.8±0.4	13.5±0.5	13.7±0.8
Lymphocytes (%)	69.4 ± 7.6	76.3 ± 5.4	66.7± 7.4	72.8 ± 6.2	68.3 ± 6.6	75.3 ± 7.3	69.6 ± 6.7	75.1 ± 6.1
Neutrophils (%)	22.3±5.5	17.2 ± 4.6	23.6 ± 5.1	16.7 ± 4.2	22.7 ± 5.0	19.3 ± 4.8	23.4 ± 4.7	18.1 ± 5.2
Eosinophils (%)	1.50 ± 0.2	1.35 ± 0.3	1.48 ± 0.4	1.41 ± 0.2	1.60 ± 0.3	1.25 ± 0.3	1.52± 0.4	1.28 ± 0.2

Values are means±SEM (n = 80, 20 per group, in each group 10 per sex).M, male; F, female

**Table 5:** Effect of the silafibrate (0, 25, 50 and 100 mg/kg) on the blood biochemical parameters of rats after 30 consecutive days of oral .administration

Parameters (units)	Silafibrate (mg/kg BW)							
	0		25		50		100	
	M	F	M	F	M	F	M	F
Glucose (mg/dL)	82.4 ± 4.53	85.5 ± 5.21	79.2 ± 2.13	83.5 ± 3.35	74.4 ± 3.11	77.5 ± 4.18	70.9 ± 2.84	75.5 ± 3.72
Triglycerides (mg/dL)	72.24 ± 3	64.56 ± 2	65.47 ± 4	58.73 ± 4	59.04 ± 3	55.42 ± 4	54.38 ± 5	50.65 ± 4
BUN	13.5±0.6	13.9±0.8	13.7±0.5	13.5±0.8	14.2±0.9	13.8±0.4	13.7±0.5	14.0±0.8
Acid Uric	69.4 ± 7.6	76.3 ± 5.4	66.7± 7.4	72.8 ± 6.2	68.3 ± 6.6	75.3 ± 7.3	69.6 ± 6.7	75.1 ± 6.1
AST(U/L)	120.6 ± 8	118.3 ± 9	126 ± 8	135 ± 6	138 ± 11	143 ± 8	159 ± 6	156 ± 9
ALT(U/L)	62 ± 6.7	65 ± 8.1	74 ± 5.2	78 ± 6.2	82 ± 7.1	80 ± 10.1	91 ± 6.7	95 ± 7.5
ALP (U/L)	148.6± 11	135.2± 9	159.6± 7	147.4± 8	166.4± 11	154.3± 9	172.1± 13	160.5± 10
Creatinine (mg/dL)	0.62±0.04	0.67±0.03	0.66±0.02	0.69±0.02	0.65±0.03	0.69±0.04	0.61±0.03	0.67±0.04
Albumin (g/dL)	3.5±0.0	3.4±0.1	3.6±0.0	3.7±0.1	3.7±0.1	3.6±0.0	3.8±0.1	3.8±0.1
Total Protein	8.1±0.1	7.8±0.1	8.3±0.1	8.1±0.1	8.4±0.1	8.0±0.1	8.6±0.0	8.4±0.1

Values are means±SEM (n = 80, 20 per group, in each group 10 per sex).M, male; F, female

## Discussion

Triglycerides and cholesterol are the most remarkable biological lipids thatplay a major role in the development of two prevalent cardiovascular risk factors, obesity and hypercholesterolemia. The dietary and pharmacological reduction of elevated plasma lipids are the two methods currently available to slow down the development of atherosclerosis. It has been estimated that every 1% reduction in LDL concentration may result in a 1% decrease in the incidence of chronic heart disease (19). Fibrates as PPARs agonists are effective in lowering elevated serum triglycerides and cholesterol (3). 

The capability of lipid lowering effect of fibrates depends on pretreatment lipoprotein status and potency of the used fibrate (20). The search for novel analogs of current drugs is a prevalent method for the discovery of novel drugs. Siliconizedanalogs of known drugs may change their properties such as chemical activity, lipophilicity, etc. This might in turn shift the interaction with a receptor and then alter the pharmacodynamics of the drug (21). These changes may alter pharmacokinetics of the new molecules and therefore also their toxicity (10). The present study demonstrated that siliconizedanalog of clofibrate, silafibrate, profoundly lowered the serum levels of total cholesterol, LDL, and triglyceride in hyperlipidemic rats. The analysis of the lipid profile data (Total Cholesterol, Triglycerides and LDL) of different groups clearly indicated that silafibrate exerted a stronger hypolipidemic activity than clofibrate.The present study also revealed that the oral treatment of hyperlipidemic rats with sliconizedanalog of clofibrate significantly enhanced the total antioxidant status (TAS) in serum and reduced MDAlevel markedly. 

The exact mechanism of antioxidant action of fibrates has remained unclear yet, and following possible routes can be proposed. First, several metabolites (not fibrates themselves) have direct radical scavenging properties (22). Second, some experiments have shown that the treatment with fibrates reduce the susceptibility of plasma proteins, especially LDLs, to oxidation (22, 23). Third, our previous studies (11, 24) and several other studies have demonstrated that fibrates possesspotent anti-inflammatory effects and antioxidant effects. Inflammatory cells are an important source of ROS generated by phagocytes› plasma membrane (25). Fibrates inhibit superoxide anion generation by leukocytes after high-fat meal in patients with ischemic heart disease (26). 

In the present study the acute and subacute toxicity of silafibrate was evaluated in mice and rats respectively. Data acquired in the acute toxicity test revealed that silafibrate was tolerated in mice up to the oral dose of 2000 mg/ kg. No mortality and other toxic symptoms were associated with the single oral administration of silafibrate in mice receiving doses up to 2000 mg /kg body weight. According to the acute toxicity test, it seems that LD50 for silafibrate is more than 2000 mg/kg in oral route. LD50 for the parent analog, clofibrate,viaoral route of administration in mice was found to be1220 mg/kg (18). Our results showed that the novel analogsilafibrate, has lower toxicity in comparison with the clofibrate in the acute toxicity test. 

In repeated-dose toxicity study up to 100 mg/ kg body weight in rats for 30-days, no toxicity or adverse effects were noted on physical examination, hematology and blood biochemical parameters indicating that silafibrate is safe when taken orally either acutely or subacutely. No deaths were recorded either in the controls or the treated rats suggesting that the LD50 for chronic oral dosing with silafibrate was much higher than 100 mg/kg per day. 

Major changes in body weight are an indicator of adverse effects of drugs and chemicals (27). In subacute oral administration of silafibrate, there were no significant changes in body weight, general behavior or food intake of the rats in the treated groups compared to the control group after the 30-day period of daily treatment. This suggests that at the chronic oral doses administered, silafibrate had no effect on the normal growth of rats. There were no significant changes in organs weight of male and female rats in silafibrate treated and control groups. In subacute study, there were slight changes in water consumption found in some treated groups,however,this change was not dose-related. The body weight or food consumption were not affected by administration suggesting that silafibrate did not induce appetite suppression and had no deleterious effects on health status, growth or development of the animals. 

The hematopoietic system, due to intensive cells proliferation, is very sensitive to toxic substances and is an important index of physiological and pathological status in man and animal (28). The hematological parameters (*i.e*. RBC, hemoglobin, WBC, MCV, MCH and MCHC) showed no significant differences between the control and the treated groups indicating that silafibrate had effects neither on the circulating blood cells nor on their production. 

The biochemical parameters (*i.e*. AST, ALT) showed slight treatment-related increases in the high dose groups (50, 100 mg/kg) as compared to the control group. Indeed, the transaminases (AST and ALT) are well-known enzymes used as sensitive markers of hepatocellular damage and within limits can provide a quantitative assessment of the degree of damage sustained by the liver (27) and as indicators showing possible toxicity (29). Generally, any damage to the parenchymal liver cells results in elevations of both transaminases in the blood (30). In addition, AST found in the serum is of both mitochondrial and cytoplasmic origin and any rise can be taken as a first sign of cell damage that leads to the outflow of the enzymes into the serum (31). Except for these parameters, there were no significant alterations in other liver function parameters such as albumin and total protein. Furthermore, there were no gross or microscopic pathological changes found in the liver. 

Creatinine is good marker of renal function (27). Any rise in creatinine levels is only observed if there is marked damage to functional nephrons (32). Renal function was measured by means of urea nitrogen and creatinine serum levels. Urea and creatinine are compounds derived from proteins which are eliminated by the kidney. In the rats that received clofibrateatdoses of 25, 50 and 100 mg/kg/day did not showurea nitrogen or creatinine levels, suggesting no alterations in kidney function. 

## Conclusion

In conclusion, the present study introduces silafibrate,asiliconizedanalog of clofibrate, and indicates that thisnew analog has greater hypolipidemic and antioxidative properties with no toxic side effects. 
